# Clinical research reactivation during the COVID-19 pandemic: An academic center process and lessons for the future

**DOI:** 10.15761/jts.1000468

**Published:** 2022-01-21

**Authors:** Jennifer Armstrong, Alison Lakin, Laurie Blumberg-Romero, Thomas Campbell, John Heldens, Christopher Lieu, Jenae Neiman, Erin Sandene, Matthew Steinbeiss, Darcy Thompson, Jason Tregellas, Thomas Flaig, Janine Higgins

**Affiliations:** 1 University of Colorado Anschutz Medical Campus, Departments of Pediatrics (Section of Neurology), Neurology, and OB/GYN (Division of Basic Reproductive Sciences), Colorado; 2 Children’s Hospital Colorado, Colorado; 3 University of Colorado Anschutz Medical Campus/University of Colorado Denver, Colorado Multiple Institutional Review Board, Colorado; 4 University of Colorado Anschutz Medical Campus, Office of Regulatory Compliance, Colorado; 5 UCHealth, Department of Research Administration, Colorado; 6 University of Colorado Anschutz Medical Campus, Department of Medicine, Division of Infectious Disease; 7 Colorado Clinical and Translational Sciences Institute, Colorado; 8 University of Colorado Anschutz Medical Campus, Department of Medicine – Division of Medical Oncology; 9 Children’s Hospital Colorado Research Institute, Colorado; 10 University of Colorado Anschutz Medical Campus, Department of Pediatrics, Colorado; 11 University of Colorado Anschutz Medical Campus, Department of Psychiatry, Colorado; 12 Research Service, Rocky Mountain Regional Veteran’s Administration Medical Center, Colorado; 13 University of Colorado Anschutz Medical Campus, Office of the Vice-Chancellor of Research, Colorado

**Keywords:** research operations, pandemic, Belmont Principles, institutional review board, clinical trials

## Abstract

**Background:**

Clinical research is a central mission of the University of Colorado Anschutz Medical Campus (CU-Anschutz). On March 18, 2020, due to rising COVID-19 rates and personal protective equipment (PPE) shortages, an emergency approval process for critical research essential to the care and safety of patients, including COVID-19 trials, was enacted. All other clinical research studies requiring face-to-face visits were placed on hold to protect participant and staff safety.

**Methods:**

A clinical research TaskForce was rapidly assembled, consisting of a cross- section of campus clinical research operations leaders, including affiliate hospitals. This group developed a guidance document and process where the primary prioritization factor was positive therapeutic benefit/risk (Groups 2-5). A REDCap form demarcating items including research visit types and safety plans was designed. A separate Space Plan Committee approval was required to gauge environmental health and safety.

**Results:**

A total of 654 protocols were approved over 31 weeks using this process. Group 2 review and approvals occurred within 5 days of campus reactivation, and 65 days after original clinical research hold. Groups 3 through 5 were opened for submission and review in a phased approach. The majority proactively submitted IRB protocol amendments to minimize face-to-face participant/staff contact. There were no cases of COVID-19 outbreak in research participants.

**Conclusion:**

Clinical research reactivation was rapidly implemented in a transparent, collaborative, broadly supported, and efficient process of staged reactivation while prioritizing the health and safety of participants and staff at CU-Anschutz. This model is practical and easily generalizable to other medical research campuses.

## Introduction

The responsibility of ensuring studies is ethical and follow the Belmont Report principles that form the framework for the Common Rule is essential in all circumstances. Cornerstones to human subject research are Beneficence, Justice, and Respect for persons [1]. Application of these principles during a rapidly changing pandemic environment is also essential yet presents distinct challenges. The Coronavirus Disease-2019 (COVID-19) pandemic posed unique risks to participants – as well as research staff – and has resulted in a shift in this benefit: risk. Within a pandemic, careful thought must be taken to provide equal access and maintenance of research opportunities to all, especially marginalized communities. Adequate information must extend to unanticipated risks to self that arise in a pandemic environment. Furthermore, it is the responsibility of the study team and, ultimately, the institution to protect those participants with diminished autonomy.

Distinction between clinical practice and research is a fundamental mainstay of research ethics. Treatments may solely be accessible through research studies for some diseases. Notable examples include cancer, rare or orphan diseases, or off-label use of FDA-approved drug/devices including pediatric administration. Research is the mechanism to provide treatment while evaluating the safety and efficacy of practice.

During COVID-19, without coordinated national guidance, institutions were forced to address this research crisis individually, not only to reduce the spread of severe acute respiratory syndrome coronavirus 2 (SARS-CoV-2) infection and conserve medical resources (e.g., personal protective equipment (PPE)), but to preserve the safety of participants, maintain research integrity, and protect study staff [2,3]. Clinical research is a central mission of the University of Colorado Anschutz Medical Campus (CU-Anschutz) and its two partner hospital systems on campus – University of Colorado Health (UCHealth) and Children’s Hospital Colorado (CHCO), Along with several specialty clinics and centers, CU-Anschutz is one of a handful of integrated health science campuses in the country. In addition, the Colorado Clinical Translational Sciences Research Institute (CCTSI), funded by the National Institutes of Health (NIH) Clinical and Translational Science Award (CTSA), provides dedicated inpatient and outpatient facilities, equipment, and personnel for conducting clinical research. The CU-Anschutz components, UCHealth, CHCO, and the CCTSI systems have independently operating research leadership infrastructures that, in a rapidly evolving pandemic environment, pose a unique challenge to enacting a coordinated response. This is compounded by shared or interdependent clinical and research space across and between hospital and campus buildings and geographic locations.

Recognizing that it was likely that at some point the pandemic would abate and allow for reopening of in-person clinical research activities, a framework was developed for a phased-approach to gradually increase, and potentially decrease, as appropriate, the volume of clinical research through a safe, swift, fair, and transparent process based on the cornerstones of Beneficence, Justice, and Respect for Persons. The framework needed to be approved by campus leadership, simple to execute across all campus entities, and transparent to study teams during this unprecedented situation.

## Materials and methods

Due to increasing statewide COVID-19 numbers, hospitalizations, and critical PPE shortages, the Governor of Colorado declared a disaster emergency on March 11, 2020 [4]. In response to this Executive Order, clinical research was placed on hold at CU-Anschutz on March 16, 2020. In-person clinical research continued in case-by-case situations when crucial for the safety of the participant or involved COVID-19 investigational treatment intervention or vaccine trials. Additionally, clinical research that could be conducted remotely by research staff (i.e., computer database studies) or use existing non-face-to-face methods (e.g., electronic surveys, phone follow-up) remained active. All other studies that did not fall into these categories and/or required face-to- face visits were placed on hold to maintain participant and staff safety and preserve critically low PPE. Approximately 250 (25%) studies remained active, a small proportion of the typically ~1000 active studies, resulting in a major campus impact, not just to participants but to the careers of many early-stage investigators.

To create and implement a process for fully reactivating clinical research on campus, the Vice-Chancellor of Research and Associate Vice-Chancellor for Regulatory Compliance assembled the Clinical Research Task Force on April 20, 2020. All clinical research locations and Centers were identified and twenty-five Task Force members, representing a cross-section of key stakeholders in campus clinical research operations at all locations were appointed, including the research directors and/or key investigators from all 16 campus clinical research centers, CCTSI leadership, and research operations and compliance personnel. This Task Force met weekly to develop a guidance document and process for clinical research reactivation. Concomitantly, working groups addressing Environmental Health and Safety, PPE Resource Utilization/Occupational Health, and COVID-19 epidemiological modeling worked to determine timing and adequate safety requirements to initiate expansion of clinical research re-entry and provided ongoing updates to the Task Force. These groups worked synergistically to identify needs and address the following principles:

### Principle #1: Follow local, state and national public health authority directives to shelter-at-home and implement social distancing

The foundation to successful clinical research reactivation hinged on providing guidance for appropriate precautions to protect all parties (participants and research staff) that was utilized in the broader geographic environment given the risk of face-to-face exposure between a researcher and a participant. Not unlike similar large regional academic medical centers, CU-Anschutz was faced with conflicting local and state guidance and jurisdictions due to its geographical and demographic boundaries amid the lack of national guidance. COVID-19 directives, although guided by the state governor’s office, were largely relegated to the purview of individual counties. Though under the administrative umbrella of the Tri-County Health Department (TCHD; Douglas, Adams, Arapahoe counties), the CU-Anschutz campus is physically located in Adams County, but straddles over six adjacent counties encompassing the metro Denver and geographic Colorado Front Range. Despite the TCHD authority, all local counties did not have complementary stay-at-home orders. This posed a challenge considering large numbers of both staff and participants would need to travel across county lines for in-person research encounters. Additionally, clinical research at CU- Anschutz includes participants from the entire hospital catchment area that encompasses seven states and, therefore, travel and local ordinances impacted individuals outside of our geographic location. CU-Anschutz, being a university campus and under TCHD, was only allowed 25% total space occupancy, regardless of the purpose of the space: clinical, research, or mixed use. UCHealth and CHCO, as hospital systems, were granted capacity and distancing exemptions by the Colorado Department of Public Health and Environment (CDPHE) which allowed these locations to open studies more quickly. UCHealth and CHCO initially developed their own internal review processes for hospital-only protocols, and were, therefore, able to activate these studies in a more expedient manner.

COVID Officials were appointed by the Associate Vice-Chancellor for Regulatory Compliance. This group represented lead principal investigators within their space or center research directors. COVID Officials met regularly to discuss and resolve barriers to campus return and served as rapid conduits of information between CU-Anschutz research leadership, Environmental Health and Safety/Occupational Health, and staff at the local level. COVID Officials developed, managed and enforced appropriate procedure for cleaning, PPE use, screening, social distancing and limited movement requirements on campus were followed within their research spaces where any face-to-face visit occurred. In some spaces, research was conducted in clinic; COVID Officials then worked in conjunction with clinic managers to ensure consistent safe practice and regulations. Given the initial 25% occupancy limitations for CU-Anschutz spaces, along with social-distancing guidance, a campus-wide space review process was created.

Detailed space plans comprised of architectural floor plans with physical distancing, traffic control, and occupancy limits explicitly delineated per space were submitted with assistance of the local COVID Official and reviewed by the Space Committee. Committee representatives were from campus Environmental Health and Safety, Occupational Health, and research administration. After initial review, all spaces were physically evaluated by Environmental Health and Safety walk-throughs with the COVID Official to ensure practicality of the plan as well as adequate ventilation.

CU-Anschutz developed electronic COVID-19 screening using REDCap that was submitted daily through OH and TCHD. Since allowances were made for essential workers at the hospitals through CDPHE as well as the individual badging access and personnel management systems, UCHealth and CHCO had independent screening and tracking processes. Therefore, all research personnel, including principal investigators, that worked in UCHealth, CHCO and CU-Anschutz spaces were initially required to complete separate screening checkpoints. UCHealth subsequently accepted CU- Anschutz wristbands for entry to decrease redundancy and administrative burden for providers and research personnel, most of whom who are CU-Anschutz employees.

All entry processes involved electronic pre-screening questionnaires based on symptoms and exposure and temperature checks at the point of entry. CU-Anschutz checkpoint entry locations were housed in key building entrances where screening approval was confirmed, temperatures were logged, and wristbands were given. These wristbands were color coded with the day of the week and dated, allowing access to any CU-Anschutz and UCHealth location regardless of checkpoint location. These checkpoint entry locations were staffed by employees that volunteered on top of their regular duties during regular business hours. Staff needing to access research space outside of regular business hours were required to complete the screening questionnaire and take their temperature at home. Participants went through similar screening methods with visitor logs in satellite research spaces to ensure 25% overall capacity. These processes allowed for detailed exposure contact tracing and decontamination in the event of an outbreak.

### Principle #2: Protect the health and safety of clinical research participants by minimizing risks as much as possible:

The Task Force worked to minimize the number of face-to-face visits needed while recognizing a level of research data integrity must be maintained. It was agreed that it was not ethical to conduct clinical research unless it was feasible to complete all research visits needed to monitor safety and obtain the data to answer the research question. Participants should not be newly enrolled if all procedures necessary to answer the primary research question could not be conducted or the participant’s safety could not be adequately monitored.

An added crucial element was to ensure the safety of vulnerable participants in the pandemic environment. CU-Anschutz conducts a variety of research involving a range of recognized vulnerable populations under the current ethical and regulatory framework including pregnant women, neonates, children, prisoners, and decisionally-challenged individuals. Participants may additionally be considered “vulnerable” due to their health status, access to clinical care, or socioeconomic situation, among other things. It was not possible to control the transportation of participants to CU-Anschutz, yet the risk of COVID-19 exposure was understood to be affected by the distance to be travelled and the mode of transportation. That risk increased further depending on the underlying condition of the participant. Unified communications by campus leadership urged principal investigators to weigh the safety of travel to campus for both staff and participants when considering requests for reactivation. Converting face-to-face procedures to remote was highly encouraged when feasible. Air travel to CU-Anschutz was discouraged unless critically essential to participant safety. A separate review process was implemented for CU-Anschutz sponsored off-campus and community- based research, which will not be discussed here.

Whenever possible, research visits were encouraged to be coordinated within clinical care visits so that participant visits to campus were reduced. The number of in-person study staff were minimized; in some cases, the PI and/or study physician conducted research tasks during standard of care visits to prevent contact with additional research personnel. Collaborations were formed between research teams, such as the biorepository groups, to employ a single collection team for multiple studies.

Additional methods identified to minimize staff and participant travel to campus included e-consent, phone follow-up, and/or electronic REDCap surveys to collect data. The rapid implementation and emergency reimbursement for clinical telehealth visits greatly aided in this endeavor to minimize face-to-face research staff contact. A vital partnership to facilitate these protocol changes was with Colorado Multiple Institutional Review Board (COMIRB). The COMIRB Director as well as a Panel Chair were included on the Clinical Research Task Force to anticipate and recommend strategies to limit face-to-face research procedures and streamline IRB reviews. For example, COMIRB issued guidance that changes to approved research needed to immediately comply with the March 16 hold on clinical research were necessary to eliminate apparent immediate hazards to the subjects and did not require prospective IRB approval. This avoided inundating the IRB with amendments and reports of protocol deviations. To augment rapid reactivation, COMIRB members, through the Director and Panel Chair, were updated on campus policies and regulatory strategies, and apprised of the urgency of pandemic-related amendment reviews. The IRBs, in turn, were responsive by holding ad hoc IRB meetings as needed and rapidly reviewing amendments, with goal turnaround time of less than 3 business days. Additionally, time-limited emergency exceptions were made to approve electronic or telehealth procedures when participant safety was at risk.

Moreover, it was widely recognized that an up-and-running, functional translational pipeline was critical to clinical research success. Basic science reactivation occurred before clinical research protocols could be considered. Screening procedures and COVID Officials were identified in these spaces, and basic science laboratories were strategically opened in a limited manner after consideration of PPE and social distancing needs. Among those, the core laboratories through the CCTSI and sample processing areas within the Clinical and Translational Research Centers (CTRCs) were swiftly evaluated and opened.

### Principle #3: Prioritize research based on the level of direct benefit to the participant

Normally, the campus has approximately 1000 active clinical trials requiring in person visits. These trials include a broad range of clinical research conducted on campus such as exploratory research, observational studies, training opportunities for junior investigators, mechanistic and translational studies focused on understanding a disease condition, as well as potentially therapeutic clinical research. The research portfolio in its entirety is important to science and society, but when prioritization was necessary, the direct benefit to participants was deemed paramount.

Working within this principle, the Clinical Research Task Force defined five broad clinical research categories (Supplement 1). Group 1 was defined as research that was critical to the clinical care and safety of patients/participants including treatment, vaccination, or clinical research for COVID-19; this Group was ongoing without hold. An additional 26 individual clinical research categories were defined to broadly encompass all clinical research at CU-Anschutz, which were ranked by all members of Taskforce via anonymous, unidentified survey into 5 reactivation Groups. Despite different research foci at each clinical research site and Center, there was near universal consensus among Task Force members for prioritization of Groups. The primary prioritization factor was positive therapeutic benefit/risk.

The Task Force acknowledged that the careers of early-stage clinical researchers and graduate students may be significantly impacted by the pandemic restrictions. It was felt critical to safeguard the future of clinical research and advancement of health-related science that was dependent on this junior investigator cohort. Therefore, early-stage investigators could have research visits prioritized within their Group but would generally not be able to conduct visits outside of their research categorization, based on participant safety.

To ensure the overall reactivation process was optimized, review of research studies requesting activity on campus by the Task Force clinical research Review Committee was required before personnel and participants were allowed campus entry. The Vice- Chancellor of Research identified a 5-person reactivation review committee with minimal conflict of interest; it was felt that a small, focused review group could more easily meet frequently, swiftly reach consensus and decrease overall review turnaround time. Led by the Associate Vice-Chancellor for Regulatory Compliance, this group included the COMIRB Director, Office of Regulatory Compliance Special Project Manager, one PhD and one MD researcher. Based on the initial REDCap research restart process developed at CHCO immediately after the shutdown, this committee along with key partner TaskForce members, IT, and research staff created a REDCap form demarcating items such as research visit types and location, PPE provisions and access to non-hospital campus buildings that investigators were required to complete, including sample management, shipping, and storage requirements. The review submission process was centralized through a campus portal with REDCap form access for all research staff. The REDCap portal additionally created a database to track reactivated protocols and facilitate rapid adaptation if there were future considerations due to the pandemic or other unexpected events. Space plan approval for each research location and COMIRB amendment approval were mandated before Review Committee approval could be granted. It was recognized that there may be a level of subjectivity in the application of the review process but the a priori group prioritization aided in making it as standardized and transparent as possible.

The Task Force and COVID Officials further recognized that there may be variations of capacity dependent on research space. Given capacity issues, different availability of PPE at different locations, and prioritization of COVID-19 studies, studies conducted only in CHCO or CTRC space did not need approval by the CU-Anschutz Review Committee and required only sequential approval by CHCO or CTRC Operational leadership followed by CHCO/CTRC Medical Director approval. Both CU-Anschutz and CHCO/CTRC review was required for protocols that utilized multiple spaces, including CU-AMC space outside of the CHCO and/or CTRC. Protocols based at UCHealth did not utilize the centralized REDCap campus portal and, instead, relied on direct approval from the Chief Medical Officer and System Director of Research Administration. This process evolved with time, as all partners recognized the benefit and equity of a single portal for submission regardless of research space. Although UCHealth continued to have a separate email submission system, they utilized the approvals from the REDCap portal to review and spearhead simultaneous approvals. The use of this centralized review portal provided a single platform for research across campus, tailoring for each partner as needed, and communication across and within sites.

A unified communication between campus and partner research leadership evolved as key to a successful process. Weekly email updates from the Vice Chancellor of Research to the broad CU-Anschutz research community were initiated at time of emergency hold, March 16, 2020. These communications were spaced to bi-weekly in August 2020 after research operations and COVID-19 numbers both stabilized. In addition, complimentary research updates by UCHealth, CHCO, and CTRC were instituted to ensure all stakeholders were reached. Finally, ongoing campus-wide and hospital-specific research town halls were organized to ensure stakeholders had access to clinical research leadership and ensure stakeholder questions could be addressed, especially surrounding reactivation submission processes. Research administration websites were restructured and reorganized to easily supply and streamline up-to-date reconstitution processes and provide direct links to review submission portals. For example, COVID-19 research pages were added to UCHealth, CHCO, and CCTSI websites with updated information on reactivation guidelines and links to the main CU- Anschutz website and submission portal.

## Results

**Figure 1.**
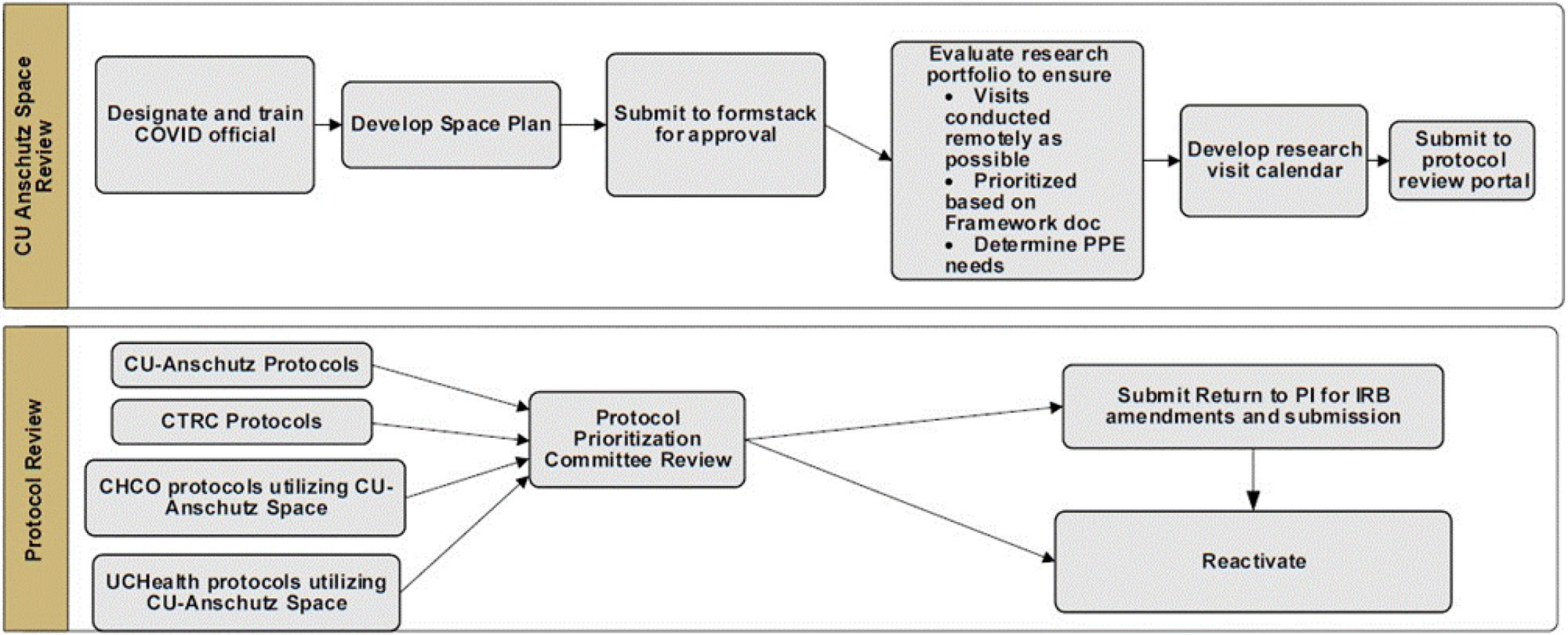
Reactivation Process for Clinical Research at CU-Anschutz

Thirty-four days passed between the initial clinical research hold and Clinical Research Task Force assembly. Campus leadership approved the Clinical Research Protocol Reactivation guidance document and prioritization groups on May 13 and the reactivation process and procedures were announced campus-wide on May 15 (Figure 1).

Task Force approval of some studies was constrained due to space, social distancing, and/or capacity limitations. This was a particular obstacle as many critical mixed clinical and research specialty areas, such as the Cancer Center, Barbara Davis Center for Diabetes, and Hemophilia and Thrombosis Center, were obligated to prioritize patient care over research visits to maintain 25% capacity. This created a disparity in research capacity issues between research studies conducted solely within hospital premises, versus studies that needed access to other campus facilities (e.g., research laboratories, research imaging facilities, administrative offices) or were exclusively contained in CU-Anschutz mixed clinical/research spaces. Therefore, social distancing capabilities and capacities varied widely. Additionally, prioritization for limited specialty inpatient and outpatient research space and resources was given to COVID-19 related research protocols considering overarching public health concerns, in alignment with University and hospital priorities.

**Figure 2.**
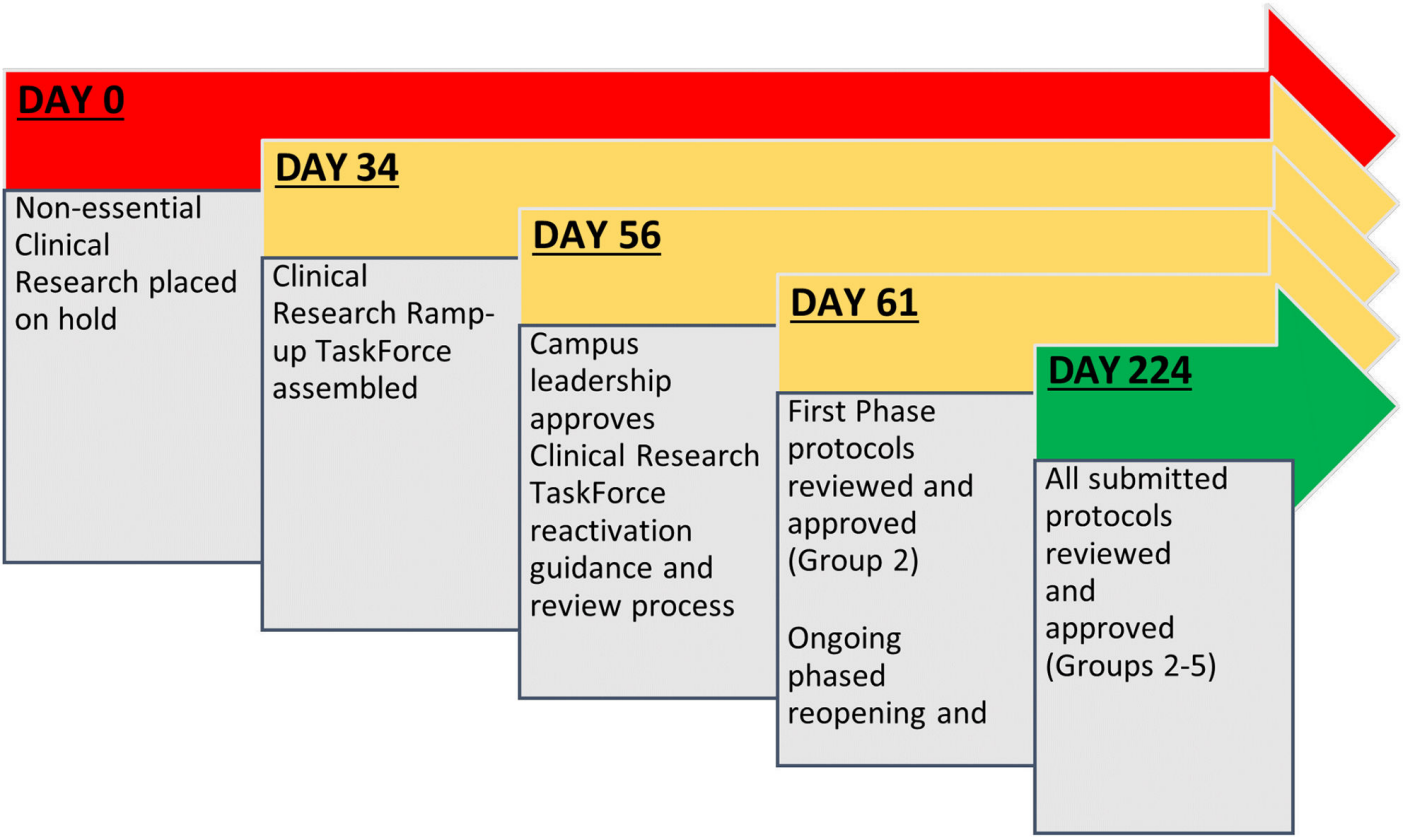
CU-Anschutz Clinical Research Reactivation Ramp-Up Timeline

Despite space challenges, a total of 654 protocols were approved over 31 weeks using this process. Group 2 review and approvals occurred within 5 days of campus reactivation, and 65 days after original clinical research hold. Over the next 5 months, Groups 3 through 5 were opened for submission and review in a phased approach taking into consideration state COVID-19 rates of infection and hospitalizations, local public health guidance, PPE capacity, and social distancing requirements per research space, with approximately each Group opening 4-6 weeks from the previous. Of the prioritization groups, Group 2 encompassed the largest collection (N=232), which were reviewed over 4 weeks. Of these, 216 were approved (93.1%). Average time to approval was around 2 business days (range 1 – 5 days); there were some outliers that took longer than 1 week due to space and protocol complexities. Turnaround time and approval rate were similar among Groups 3, 4, and 5 (Figure 2).

A minority of protocols (4.4%) had their self-identified group reclassified by the Clinical Research Review Committee during the review process, with review committees assigning these studies a higher group number, based on protocol specifications.

**Figure 3.**
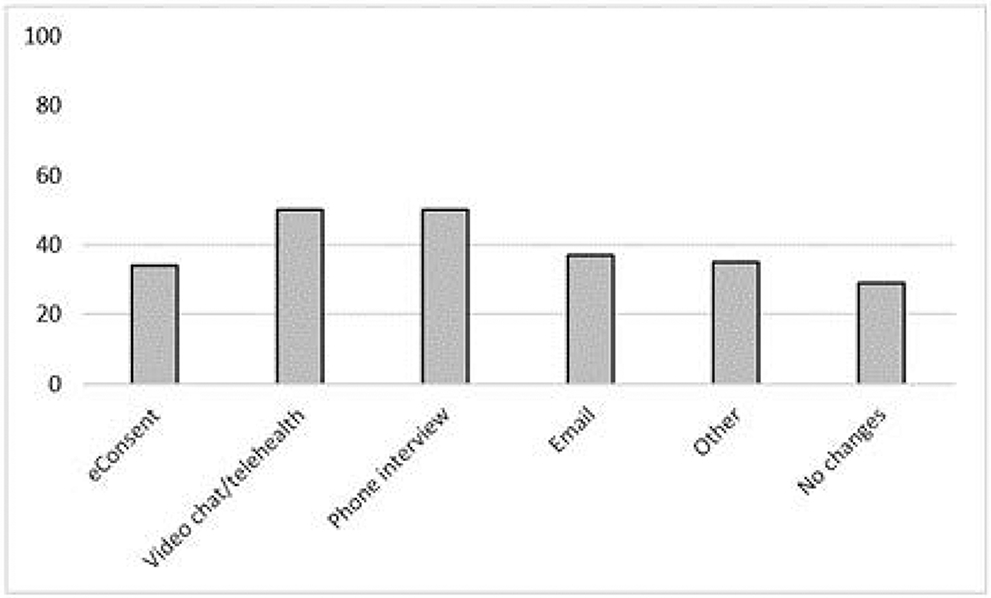
Percentage of IRB Protocol Amendments Employed During CU-Anschutz Clinical Research Reactivation

Of all approved protocols, the majority (78.7%) proactively submitted COMIRB protocol amendments to minimize face-to-face participant/staff contact (Figure 3).

Amendments included telehealth/video visits (55.7%), phone interviews (53.7%), email follow-up (40.6%), and eConsent (34.7%). Most protocol amendments went to expedited COMIRB chair review with typical submission to approval time within 2 business days (range 0-3 days).

The majority of protocols involved biospecimen collection that required access to campus areas outside the collection facility (e.g., transport to wet labs for specialized assays; N = 544, 80.0%). Access to campus laboratory space or administrative offices accounted for over two-thirds of non-hospital facility requests (69.3%). Access to the inpatient (N= 155; 23.9%) and outpatient CTRC (N=304; 46.8%) space together similarly accounted for most space requests (70.7%). There was significant overlap across requests; typically, protocols required access to multiple spaces on campus including the primary site of research. A minority (N=90; 14.3%) did not request new patient enrollment but requested the resumption of research related visits or access to administrative offices to continue or complete research projects. These requests tended to be for protocols in Groups 4 and 5.

COVID Officials were integral in determining minimal staff numbers and exposure for adequate research coverage, ensuring staff followed checkpoint screening, social distancing and PPE regulations. COVID Officials also worked collaboratively to monitor and accommodate research staff requiring access to their respective facilities across campus spaces. For the 654 approved protocols, there was an average of 2 research personnel and 39 research participants per study requiring campus facility access. From a safety standpoint, there were no reactivated studies associated COVID-19 outbreaks (defined as 2 individuals testing positive within a shared campus space). No individual participants or staff contracted SAR-CoV-2 related to face-to-face research visits. Daily space logs confirmed 25-50% occupancy spacing as designated for the space and compliance in PPE requirements. In hospital-associated clinical research areas with greater flexibility and not under occupancy limits, there again were no research related SAR-CoV-2 infections in participants or staff. No research visits or protocols were halted once reactivated due to non-compliance or exposures. Finally, there were no critical shortages of PPE on campus affecting either clinical or research settings with the ramp-up of campus research operations.

## Discussion

It is incumbent upon the institution to protect research participant interests in a safe and practical manner. The unprecedented COVID-19 situation required an unbiased, transparent framework to gradually increase, and potentially decrease during subsequent surges, the volume of clinical research on campus through a fluctuating safety lens. The CU-Anschutz Clinical Research Task Force rapidly implemented a transparent, broadly supported, efficient process of staged clinical research reactivation while prioritizing the health and safety of participants and staff. The Clinical Research Review committee reviewed and reopened the majority of clinical research with rapid turnaround. Concurrent space planning and IRB reviews of protocol amendments augmented rapid approval and ensured that all studies had appropriately adapted to the new safety environment of the pandemic.

The critical foundation for success was having representation and communication at inception from all major areas where clinical research was conducted. This process established a valuable campus research leadership working group that will persist beyond the pandemic. Representation not only created a relatively research inclusive process but aided in communication and buy-in between spaces. On a larger scale, unified email communications and realtime consolidated websites ensured consistent and efficient messaging. However, the process was not without its issues. Given the rapidly changing environment, email fatigue was experienced. Restrictions were made by the campus communications department on order and timing of email announcements. Email composition and website branding and structural limitations by CU-Anschutz, UCH, and CHCO added difficulty for user navigation. A communication process from a single central research leadership entity representing CU-Anschutz and its partners would alleviate many of these matters. This central communication method was ultimately employed. The Clinical Research Task Force meets weekly or bi-weekly, as needed, and prepares a joint statement for distribution in the regularly scheduled Vice Chancellor Research Updates emails and Associated websites are updated concomitantly with the email communication.

This spirit of collaboration continued to grow beyond the committee. When possible, COVID Officials and study teams made available spaces with more flexible accommodations and/or consolidated research personnel/procedures for other teams. This was of particular importance for junior investigators, fellows, and doctoral students to harness collaborative means of data collection and integrity. The IRB additionally met with study teams to brainstorm creative means of non-face-to-face methods to keep research going as much as possible. Ongoing communication to COMIRB staff and reviewers helped further facilitate rapid amendment turnaround and minimize gaps in data collection.

In line with the mission of an agnostic and safe reactivation approach, the small, focused submission review committee ensured a dedicated and efficient team with minimal conflict of interest that was responsive, attentive, and available for questions and rapid review. Aiding in the review process was the submission portal, which safeguarded a rapid, uniform and transparent platform across and within partners. A single platform additionally permitted tracking of submitted and approved protocols for reference. The REDCap platform afforded a rich database to aid in research related activities. The database helped track reactivation and provided a way for non- centralized services to know if a protocol was approved by the relevant entities and what group it fell into. This was helpful determine research procedure scheduling prioritization based on space and resource capacity, track daily space and staff access and ensure only approved studies were active.

While focused on rapid creation and implementation of the reactivation process in March 2020, we did not proactively outline a process to establish research roll- back/pause plan for COVID-19 resurgence and/or more restrictive public health orders. In retrospect, we recognized that classifying protocol by subgroup rather than group (e.g., 3b instead of 3) from the start was essential for providing granularity the event of surge. Although the review committee could readily determine subgroup from the information provided through the portal, this decreased efficiency and created extra work on the backend when faced with worsening statewide COVID-19 numbers in November 2020 [5].

Noting there would be some allowances due to space flexibility, studies were opened for review and reactivation in a grouped and staged approach in accordance with local and state public health guidelines. COVID officials, combined with a rigorous return to campus procedures, allowed tracking of all staff access, daily census generation, and contact tracing vehicle if needed. These logs were available to CDPHE and TCHD upon request to preserve compliance. It was recognized that, despite best efforts, there were some difficulties for staff (typically physicians) who went between hospital and non-hospital spaces. Instances occurred where a COVID exposure while wearing PPE did not prevent working in the hospital but prevented one from accessing CU-Anschutz research space. The default was the most stringent of the recommendations, which placed strain on clinical staffing and forced some staff to utilize vacation time while asymptomatically self-quarantining. This caused confusion and strain among staff.

Despite best efforts to be viewed as a cohesive health care campus system and recommend consistent guidance there as, this was ultimately limited by the authority of TCHD. A cohesive screening process with associated consistent guidelines would have both cause less shortage of clinical and research staff and permitted less interruptions to active studies.

In the context of the Belmont Principles, we were able to create and implement a process that kept both participants and research personnel safe while maintaining low COVID-19 transmission. The guiding pilar of Beneficence ensured harm was minimized with considering PPE/safety, local and state guidance, and using a grouped and staged process, while still maximizing benefit: risk ratio and reactivation urgency. Additionally, participants and staff were fully informed of expectations of revised research visits, providing access to ongoing treatment and safety visits. While striving for Justice by creating disease agnostic groups and instead focusing on therapeutic intent and space utilization, we recognized there were areas for improvements. Expanding telehealth capabilities, infrastructure, and remote clinical research partnerships especially in marginalized, rural or out of state communities would equalize the access of distribution and fairness for those participants to either gain access to clinical studies or maintain safety procedures. This was a huge gap, and an area for ongoing legislation and advocacy. Finally, Respect for Persons was essential to the success and maintenance of reactivation. By reactivating in a swift and safe manner, respect for persons was afforded to complete the study from start to finish, while apprising participants and staff of risk of travel to campus and on campus safety guidelines. Participants could continue or withdraw in studies – and staff were allowed to work remotely – without penalization. Additionally, by allowing pandemic-related protocol deviations for COMIRB to be lumped into the continuing review document, study teams did not undergo undo work or adverse repercussions.

Despite lessons learned, we were able to increase clinical research activity in a safe, phased, systematic approach for all research participants, staff, and the entire CU- Anschutz campus regardless of whether individual research activities occur at UCHealth, Children’s Hospital Colorado, or CU-Anschutz clinic/research facility space, in the context of the ongoing COVID-19 pandemic.

## Supplementary Material

Supplement 1
