# Supplementary material for: Clinical research reactivation during the COVID-19 pandemic: An academic center process and lessons for the future
Source: J Transl Sci. Author manuscript; Available in PMC 2023 Apr 27. (PMC10139738; doi:10.15761/jts.1000468)
Supplement: Supplement 1 [file NIHMS1816090-supplement-Supplement_1.pdf]

Principles and Framework

Guiding a Phased Approach to Ramping up On-Campus Clinical Research Activity

CU Anschutz Medical Campus

GOAL: To increase clinical research activity in a safe, phased, systematic approach for ALL research participants, staff, and the entire CU Anschutz campus regardless of whether individual research activities occur at UCHealth, Children's Hospital Colorado, or CU clinic/research facility space, in the context of the ongoing COVID-19 pandemic.

The COVID-19 pandemic is unpredictable and expected to remain in the months if not years ahead. Clinical care has fundamentally changed for the foreseeable future and we must consider how clinical research can safely and effectively operate in this new reality. COVID-19 poses unique risks to participants and research staff that need to be addressed as clinical research cannot occur without the partnership of participants and their willingness to agree to follow a specific research protocol. The responsibility to ensure that studies are ethical and follow the Belmont principles that form the framework for the Common Rule are important in normal circumstances but become even more so in this uncertain and rapidly changing environment. The cornerstones to human subject research are beneficence, justice, and respect for persons. Beneficence involves protecting the well-being of research participants by maximizing the benefits of research and minimizing any risks. COVID-19 poses unique risks to participants and research staff, and has resulted in a shift in the risk : benefit ratio.

Given the broad range of clinical research on campus, each Principal Investigator has a significant responsibility to consider these principles when making a decision as to the appropriate environment under which their study should be re-activated. Furthermore, it is incumbent on the institution to protect research participant interests in a safe and practical manner. These uncertain times are likely to extend into the future. The most appropriate way to continue clinical research during this time is to conduct as many visits as possible remotely via telehealth or phone visits. The expectation is that as many screening, enrollment and research visits are conducted remotely whenever possible. However, for some clinical research key face to face visits are essential for research procedures and safety.

In the current state, in-person clinical research continue in situations where it is interconnected with necessary clinical care, provides investigational product to COVID-19+ patients, or critical for the safety of the participant. As of April, 2020, approximately, 250 studies are currently active on campus; this is a small proportion of the clinical research portfolio that normally includes over 5000 active clinical research projects. Unfortunately, it is unlikely that all in-person clinical research activities will be able to continue for a significant period of time. This unprecedented situation requires a framework to gradually increase, and potentially decrease as appropriate, the volume of clinical research on campus.

This phased-approach framework is informed by the following principles and priorities. Additional guidance is provided given the unique circumstance within an academic research framework:

Objective: Implement a fair and transparent process for approving clinical research to occur on campus

ACTION: Develop a clear, equitable and transparent process. This process should be easy for clinical researchers to follow while allowing fair and adequate representation of the above principles.

ACTION: Approve this process. University and affiliate hospital leadership approval will be required before implementation.

ACTION: Implement this process. To ensure this process is optimized, review of research studies requesting activity on campus by the Taskforce Clinical Research Review Committee *is required* before a study can obtain any regulatory approvals, if needed. It is recognized that there may be a level of subjectivity in the application of this process but it will be standardized as much as possible.

ACTION: Track active protocols to allow rapid adaptation to a changing environment through effective communication if there are future considerations due to the pandemic or other unexpected events.

Principle #1: Follow the cognizant Local, State and National Public Health Authority directives to shelter-at-home and implement social distancing:

PRIORITY: To provide guidance for appropriate precautions to protect all parties (participants and research staff) that is *at least at the same level* utilized in the broader clinical environment given the risk of face-to-face exposure between a researcher and a participant.

PRIORITY: To manage the research environment where any face-to-face visit occurs, including to ensure that the appropriate cleaning, PPE, screening, hand washing/sanitizing, social distancing requirements and limited movement on campus are followed.

PRIORITY: To help ensure the safety of vulnerable participants in this current environment. This university conducts a variety of research involving a range of recognized vulnerable populations under the current ethical and regulatory framework including: pregnant women, neonates, children, prisoners and decisionally-challenged individuals. Patients may additionally be considered vulnerable due to their health status, access to clinical care, or socioeconomic situation, among other things. It is not possible to control the transportation of participants to this campus. The risk of exposure is affected by the distance to be travelled and the mode of transport used. The risk increases further depending on the underlying condition of the participant. The Principal Investigator must take these additional risks into consideration before requesting that participants come to campus for their study.

Principle #2: Protect the health and safety of our clinical participants by minimizing risks as much as possible.

PRIORITY: To continue to conduct ethical, safe, human subject research. It is not ethical to conduct clinical research unless it is feasible to complete all the essential research visits needed to monitor safety and obtain the essential data points to answer the research question.

Participants should not be enrolled if all procedures necessary to answer the primary research question cannot be conducted or the participant's safety cannot be adequately monitored.

PRIORITY: Whenever possible research visits should be coordinated within clinical care visits so that participant visits to campus are reduced.

PRIORITY: To minimize the number of visits needed on campus while recognizing that the value of clinical research is in the high quality of data obtained. This strategy includes, but is not limited to: submit amendments to the protocol, consent and contract to codify the modifications needed to use e-consent, telehealth visits, and/or redcap surveys to collect data or phone visits.

Principle #3: Research restart should be prioritized based on the level of direct benefit to the participant. Normally, the campus has approximately 1000 active clinical trials requiring in person visits. These trials include a broad range of clinical research conducted on campus including exploratory research, training opportunities for junior investigators, mechanistic and translational focused on understanding a disease condition, as well as clinical research that is potentially therapeutic. The research portfolio in its entirety is important to science and society, but when prioritization is necessary the direct benefit to participants is paramount.

PRIORITY: To further differentiate potentially therapeutic intent studies:

- Essential research that is critical to the clinical care and safety of patients/participants including access to treatment or clinical research for COVID-19+ patients – *currently occurring*
- Studies with therapeutic intent when no standard of care treatment is available
- Studies with therapeutic intent but standard of care is a viable option
- Studies with therapeutic intent that compare two or more standard of care options
- Studies with prevention strategies to minimize disease progression

Guidance #1: Protect the careers of early stage clinical researchers

PRIORITY: To protect the next generation of clinical researchers. Careers of early stage clinical researchers may be significantly impacted by the current environment. Already, the number of clinical researchers is declining. Therefore, it is critical to safeguard the future of clinical research and advancement of health-related science dependent on this particular investigator cohort. Early stage investigators may have research visits prioritized within the phase that their research encompasses (Table 1) but will generally not be able to conduct visits outside of their research category, which is based on participant safety.

Guidance #2: Graduate students are students first, researchers second

PRIORITY: To maintain student safety and academic goal focus by sheltering-at-home. Except under the most exceptional of situations, clinical research visits should be conducted by clinical research faculty or staff. Graduate students may be considered to aid in clinical research projects where their role can be conducted remotely (i.e. redcap data entry, telephone consent).

Priority Ranking of Clinical Research by Grouping:

**Group 1**	Essential research that is critical to the clinical care and safety of patients/participants including access to treatment or clinical research for COVID-19+ patients and HCW<br>Complete active visits<br>New enrollment in currently active study<br>Open new study

**Group 2**	Complete visits for enrolled participants to studies with therapeutic intent when no standard of care treatment is available;<br><br>New enrollments to a currently active study with therapeutic intent when no standard of care treatment is available;<br><br>Complete visits for enrolled participants to studies with therapeutic intent but a standard of care is a viable option;<br><br>Open a new study with therapeutic intent when no standard of care treatment is available;<br><br>Complete therapeutic or non-therapeutic protocols that require on campus processing but no participant contact (includes large data set analysis, processing of specimens);<br><br>Complete visits for enrolled participants to studies with prevention strategies to minimize disease progression (e.g. screening for cancer, use of medication to prevent disease progression, birth control, use of supplements);<br><br>Complete visits for enrolled participants to observational protocols involving minor research interventions that can be conducted during SOC visits ("Minor" is defined as low risk procedures such as blood draw, additional radiology scan, diet, OGTT)

**Group 3**	New enrollments to a currently active study with therapeutic intent but a standard of care is a viable option;<br><br>New enrollments to currently active therapeutic or non-therapeutic protocols that require on campus processing but no participant contact (includes large data set analysis, processing of specimens);<br><br>New enrollments to a currently active study with therapeutic intent that compare two or more standard of care options;<br><br>New enrollments to a currently active study with prevention strategies to minimize disease progression (e.g. screening for cancer, use of medication to prevent disease progression, birth control, use of supplements);<br><br>New enrollments to a currently active study for observational protocols involving minor research interventions that can be conducted during SOC visits ("Minor" is defined as low risk procedures such as blood draw, additional radiology scan, diet, OGTT);<br><br>Open a new therapeutic or non-therapeutic protocol that require on campus processing but no participant contact (includes large data set analysis, processing of specimens);<br><br>Complete visits for enrolled participants to non-therapeutic protocols requiring SOC and additional complex research procedures and/or visits (participants with underlying condition where some visits are clinical but there are also higher risk research only procedures such as PK draws, PET CT, MRI, DEXA, biopsies, exercise testing, insulin clamps, calorimeter room);<br><br>Complete visits for enrolled participants to non-therapeutic protocols requiring minor research only interventions (includes normal health participants as well as participants with underlying condition for research only such as blood draw, additional radiology scan, diet, OGTT).

Group 4	Open a new study with therapeutic intent but a standard of care is a viable option<br><br>Complete visits for enrolled participants to non-therapeutic protocols requiring complex research only interventions, (includes normal health participants as well as participants with underlying condition for series of tests as well as higher risk research only procedures such as PK draws, PET CT, MRI, DEXA, biopsies, exercise testing, insulin clamps, calorimeter room);<br><br>Open a new study with therapeutic intent that compares two or more standard of care options;<br><br>New enrollments to currently active non-therapeutic protocols requiring minor research only interventions (includes normal health participants as well as participants with underlying condition for research only such as blood draw, additional radiology scan, diet, OGTT;<br><br>Open a new study for prevention strategies to minimize disease progression (e.g. screening for cancer, use of medication to prevent disease progression, birth control, use of supplements);<br><br>New enrollments to a currently active study for non-therapeutic protocols requiring SOC and additional complex research procedures and/or visits (participants with underlying condition where some visits are clinical but there are also higher risk research only procedures such as PK draws, PET CT, MRI, DEXA, biopsies, exercise testing, insulin clamps, calorimeter room).

**Group 5**	Open a new observational protocol involving minor research interventions that can be conducted during SOC visits (“Minor” is defined as low risk procedures such as blood draw, additional radiology scan, diet, OGTT);<br><br>New enrollments to currently active non-therapeutic protocols requiring complex research only interventions, (includes normal health participants as well as participants with underlying condition for series of tests as well as higher risk research only procedures such as PK draws, PET CT, MRI, DEXA, biopsies, exercise testing, insulin clamps, calorimeter room);<br><br>Open a new non-therapeutic protocol requiring SOC and additional complex research procedures and/or visits (participants with underlying condition where some visits are clinical but there are also higher risk research only procedures such as PK draws, PET CT, MRI, DEXA, biopsies, exercise testing, insulin clamps, calorimeter room);<br><br>Open a new non-therapeutic protocol requiring minor research only interventions (includes normal health participants as well as participants with underlying condition for research only such as blood draw, additional radiology scan, diet, OGTT;<br><br>Open a new non-therapeutic protocol requiring complex research only interventions, (includes normal health participants as well as participants with underlying condition for series of tests as well as higher risk research only procedures such as PK draws, PET CT, MRI, DEXA, biopsies, exercise testing, insulin clamps, calorimeter room.
